# Efficiency of Kolmogorov–Arnold Networks in Small Medical Samples (Case Study of 2D Brain MRI Image Segmentation)

**DOI:** 10.17691/stm2026.18.2.01

**Published:** 2026-04-30

**Authors:** G.Yu. Manzhos, I.V. Tomilov, V.V. Goncharov, K.S. Yashin

**Affiliations:** PhD Student, Faculty of Applied Informatics; ITMO University, 49, Bldg. A, Kronverksky Prospekt, Saint Petersburg, 197101, Russia; Teaching Assistant, Information Technology Department; Privolzhsky Research Medical University, 10/1 Minin and Pozharsky Square, Nizhny Novgorod, 603005, Russia; Senior Laboratory Technician; ITMO University, 49, Bldg. A, Kronverksky Prospekt, Saint Petersburg, 197101, Russia; PhD, Associate Professor, Information Technology Department; Privolzhsky Research Medical University, 10/1 Minin and Pozharsky Square, Nizhny Novgorod, 603005, Russia; MD, PhD, Associate Professor, Department of Traumatology, Orthopedics, and Neurosurgery; Privolzhsky Research Medical University, 10/1 Minin and Pozharsky Square, Nizhny Novgorod, 603005, Russia; Neurosurgeon, Neurosurgery Department, University Clinic; Privolzhsky Research Medical University, 10/1 Minin and Pozharsky Square, Nizhny Novgorod, 603005, Russia

**Keywords:** deep learning, computer vision, Kolmogorov–Arnold networks, segmentation, KAN, U-Net

## Abstract

**The aim of the study** was to evaluate the efficiency of the KANU-Net 2D architecture based on U-Net in medical segmentation tasks of 2D brain MRI images on BraTS dataset with a limited number of training samples.

**Materials and Methods:**

The experiments were carried out using the subsamples containing 50, 100, and 150 images. The study described the data preprocessing steps, including normalization, gamma correction, cropping, and augmentation. A combination of Dice loss and BCE loss was used as a loss function. The network was optimized using AdamW. The network operation performance was evaluated using Accuracy and Dice coefficient for each region and its mean Dice.

**Results:**

KANU-Net 2D was experimentally demonstrated to achieve competitive performance comparable to current SOTA models of convolutional neural networks when trained on small samples. Specifically, the mean Dice coefficient reached 0.851 when using 100 training samples.

**Conclusion:**

The conducted studies showed KANU-Net 2D network to outperform the Med-DANet segmentation model both in terms of a mean value and region classes. The model effectiveness for different tumor regions highlighted the ability of the KAN-based (Kolmogorov–Arnold network) approach to adapt to various image characteristics in medical segmentation tasks. The obtained results demonstrated the undeniable promise of applying KAN for medical image segmentation in small samples and can lay the foundation for further research in this field.

## Introduction

Tumor segmentation in brain MRI images is a daunting challenge for digital medical data processing. Early detection and diagnosis play a critical role in effective treatment. According to WHO, in 2020, nearly 10 million people died from cancer worldwide [1]. In contrast to screening X-ray of the lungs [2], according to the study [3], there are no such examinations for brain, and cancer markers detected in patients fail to localize tumors in routine examinations [3]. Images taken using different MRI modalities have different settings of the parameters: brightness, gamma, etc. [4], and current computer image processing algorithms cannot effectively adapt to any MRI examinations.

**Figure 1.**
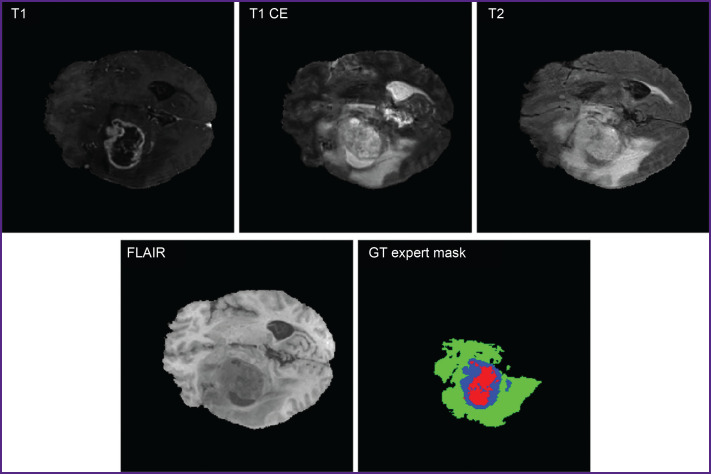
Brain MR image in different modalities (T1, T1 CE, T2, FLAIR) and color segmentation (GT expert mask) of three classes: necrotic and non-enhancing tumor core (NCR/NET — red), peritumoral edema (ED — green) and enhancing tumor (ET — blue)

Current segmentation techniques of medical images include threshold segmentation, region split and merge method, morphological segmentation, clustering methods, region growing technique, etc. [5, 6]. However, not infrequently, obtained MR images are analyzed by doctors in a manual way, which is a resource-consuming and complex process [7]. Figure 1 demonstrates MRI images in different modalities (T1, T1 CE, T2, FLAIR [3]) and GT expert mask indicated by medical experts.

Currently, the algorithms based on ultraprecise neural networks appear to be advancing techniques for medical image segmentation [8]. U-Net can serve an example of such algorithm [9], it has been proved as one of the best architectures to work with medical images. Many other modern techniques were developed based on this network.

U-Net includes two main parts: an encoder and a decoder. The first is responsible for image convolving into a minor pixel form, while the latter, on the contrary, increases the resulting image resolution. These two parts are interconnected by convolutional layers, which realize the algorithms of extracting key signs at different abstraction levels. Each encoder layer is responsible for searching certain pixels in an image and for generating the sign map; and the decoder layer provides the discretization increase of the resulting sign map. The next stage involves such operations as batch normalization and a nonlinear function of ReLU activation followed by one more convolution and maxpooling, 2×2 in size, which decreases spatial dimensions of the obtained feature matrix, thereby crunching the information for the following passage to the network. In the network output we can get the probability of the image belonging to one of the predetermined classes.

The analysis of works with the models based on U-Net 2D architecture [10–12] showed the high values of Dice coefficient (the main metric of the network quality) to be achieved at large training sample volumes — 369 images and more. Since modern models of neural networks require giant datasets for training, there is a problem in making algorithms operating on small samples with the same efficiency [13].

Small samples appear by the reason that in medicine there are rare diseases, and due to this fact, there are small amounts of observations. As a result, we cannot apply serious projects based on the models of convolutional neural networks for real-world practical problems [14]. In neuro-oncology the problem of small samples is particularly topical for rare brain tumors or the tumors of uncommon location. The development of means capable to effectively operate under the conditions of low quantity data will enable to accelerate the implementation of clinical decision support systems into routine clinical practice.

Neural networks based on Kolmogorov–Arnold theorem (KAN) [15] are promising considering their applicability on small samples. Among their advantages reported in literature [16] there are the following ones: resilience when training using small data, although for medical data the approach is not adequately studied. The low learning rate is mentioned as one of the disadvantages. The reviews of other KAN characteristics are represented in literature sources [15, 16].

At present, the current studies on KAN in medicine are underreported, despite the fact that KAN has proved to be promising in small medical samples. Additionally, the existing studies primarily have focused on classification and simple pictures with high contrast distinguishability, such as MedMNIST (large-scale collection of standardized medical images) and chest X-ray with clearly visible features [17, 18] that prevents from covering the whole range of tasks based on medical image processing.

The aim of the study was to evaluate the efficiency of the KANU-Net 2D architecture based on U-Net in medical segmentation tasks of 2D brain MRI images on BraTS dataset with a limited number of training samples.

## Materials and Methods

### The network architecture

The study used KANU-Net 2D network [19] based on a classic U-Net with encoder-decoder structure and residual connections. In this architecture the standard Conv2D convolutional layers were replaced with FastKANConvLayers. Each FastKANConvLayer included the conversion of input signs using radial basis function (*RBF*):

RBF(x,y)=exp−∥x−y∥22σ2,

where *x* — input image vector, *y* — function center, σ — kernel width.

**Figure 2.**
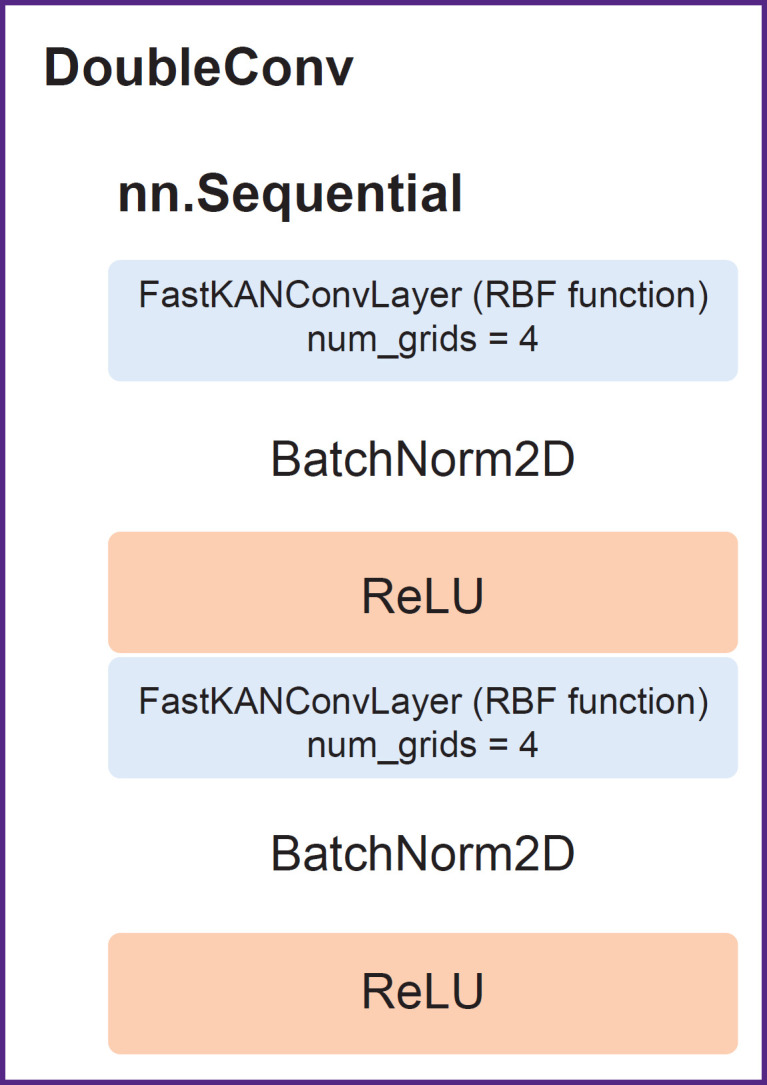
DoubleConv with built-in layers FastKANConvLayer

The network involved an encoder — a series of DoubleConv (Figure 2) (two successive layers FastKANConvLayers followed by batch normalization and ReLU activation) and a decoder.

The network processed 2D sections with 4 input channels, which corresponded to four modalities, and provided a four-channel segmentation mask representing the probability distribution of anatomical structures.

### Dataset

A benchmark dataset BraTS 2020 was used as batch data [20], from which randomly there were extracted 3 training subsamples, their volume being 50, 100, 150 images. A four-channel image from the dataset consisted of several modalities: T1 — weighted image; T1 CE — contrast (gadolinium) enhanced weighted image; T2 — weighted image; FLAIR — fluid attenuated inversion recovery.

Moreover, each image had annotations indicating tissue type verified by 4 clinical experts: healthy tissue; peritumoral region/edema region–infiltration; non-contrast core; necrotic core; enhancing tumor region.

The experiments involved 2D sections extracted from 3D data with the focus on axial sections most accurately presenting tumor regions. The present segmentation enables to distinguish key regions of interest according to BraTS standards by three classes: whole tumor (WT); tumor core (TC); enhancing tumor (ET).

### Data preprocessing

A preprocessing technique used in the present study involved several stages to improve the quality and homogeneity of input data:

1. each MRI modality was independently normalized in the range [–1.0; 1.0] using min–max scaling to standardize the distribution of intensities to different scans;
2. gamma-correction, gamma values being 1.0, 1.5, 1.5, 1.2 for T1, T1 CE, T2 и FLAIR sections, respectively;
3. the images were cropped to a fixed size 224×224 pixels, and centered to the brain regions.

When forming a training dataset, we used data augmentation, which included occasional 90° rotations and occasional shifts to improve the model generalization capabilities.

### Loss function and optimization

As a loss function (*Loss*) we chose the combination based on Dice loss and binary-cross entropy (*BCE loss*), which considered significant imbalance of classes in segmentation:

Loss=(α⋅BCE loss++ (1−α)⋅Dice loss).

The value ɑ=0.2 was found empirically to provide the training stability and optimal quality of the resulting segmentation.

BCE loss=−1N∑i=1Nyi⋅log(pi)++ (1−yi)⋅log(1−pi),

where *N* — the number of pixels in an image; *y_i_* — true mark (0 or 1) of *i* pixel; *p_i_* — the probability of *i* pixel belonging to class 1 predicted by the model; log — natural logarithm.

Dice loss=1−2ΣipigiΣipi+Σigi,

where *p_i_* — a predicted value for *i* pixel; *g_i_* — true mark (0 or 1).

As an optimizer, we used AdamW with the initial learning lr=1e–4 and weight decay 1e–6. The scheduler chose “cosine annealing” with restarts and minimal learning rate 1e–3.

### Evaluation metrics

The study carried out a complex assessment including Accuracy metrics, mean coefficient Dice and Dice coefficient for regions (*Dice_region_*): WT tissues, TC and ET. Mean Dice calculated as the mean value for Dice coefficients for ET, TC and WT served the main metric to choose the best model.

Diceregion=2|Xregion∩Yregion||Xregion|+|Yregion|,

Accuracy=TP+TN(TP+TN+FP+FN),

where *TP* (true positives) — the number of true positive results; *TN* (true negatives) — the number of true negative results; *FP* (false positives) — the number of false positive results; *FN* (false negatives) — the number of false negative results.

### Experiments

The experimental procedure consisted in KAN network training independently on three subsamples with different volumes and comparing the findings with the existing SOTA models of convolutional neural networks.

The experiments were carried out using the following configuration:

frameworks: pytorch, monai [21];
the sizes of training samples were 50, 100, 150 images;
the sizes of validation and test samples were 50 images;
batch size — 2;
4 grid points in KAN (num grids);
[−1.0; 1.0] the range of KAN network grids (grid min, grid max);
number of epochs — 100.

## Results

Tables 1 and 2 represent the main performance metrics of KANU-Net 2D model on a test set BraTS 2020, focusing on specific tumor regions (ET, WT, TC). Table 3 demonstrates the comparison of the developed approach efficiency with other popular models on the same dataset, but with a larger training sample.

**T a b l e 1:** Comparison of KANU-Net 2D model performance for different sample sizes

Number of images	Accuracy (%)	ET Dice (%)	WT Dice (%)	TC Dice (%)	Mean Dice (%)
50	0.993	0.801	0.858	0.797	0.819
**100**	**0.993**	**0.812**	**0.899**	**0.843**	**0.851**
150	0.990	0.673	0.853	0.806	0.777

N o t e: ET — enhancing tumor, WT — whole tumor, TC — tumor core.

**T a b l e 2:** Comparison of KANU-Net 2D model performance for the classes: necrotic and non-necrotic tumor core (NCR/NET), peritumoral edema (ED), and enhanced tumor (ET)

Number of images	NCR/NET Dice (%)	ED Dice (%)	ET Dice (%)
50	0.766	0.818	0.801
**100**	**0.778**	**0.844**	**0.812**
150	0.638	0.719	0.673

**T a b l e 3:** KANU-Net 2D and SOTA models compared

Model	Number of images	ET Dice (%)	WT Dice (%)	TC Dice (%)	Mean Dice (%)
Med-DANet [10]	369 (train), 125 (val)	0.805	0.902	0.813	0.840
nnU-Net [11]	369 (train), 125 (val)	0.794	0.911	0.852	0.852
H2NF-Net [12]	369 (train), 125 (val)	0.827	0.887	0.853	0.855
**KANU-Net 2D**	**100 (train), 50 (val)**	**0.812**	**0.899**	**0.843**	**0.851**

N o t e: train — training sample, val — validation sample, ET — enhancing tumor, WT — whole tumor, TC — tumor core.

**Figure 3.**
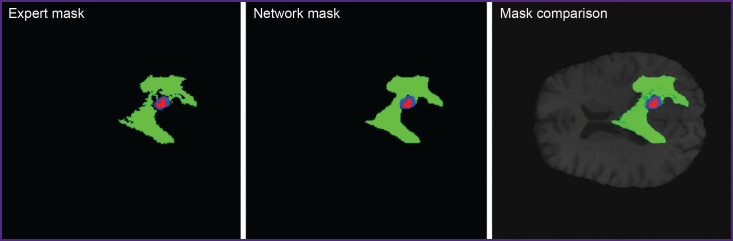
Expert segmentation mask, KANU-Net 2D network prediction mask, and their comparison in one MR image

The experiments showed KANU-Net 2D model trained on small samples to achieve the mean Dice coefficient equal to 0.851. The findings appeared to exceed the model purposely developed for medical segmentation Med-DANet [10], and comparable with current SOTA models trained on full dataset BraTS 2020. Figure 3 represents the imaging findings.

**Figure 4.**
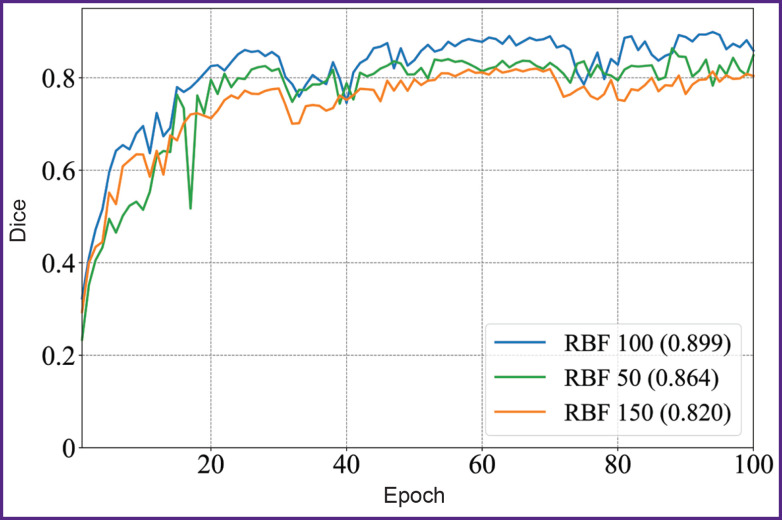
Diagram of the mean Dice coefficient depending on the epochs in KANU-Net 2D network (50, 100, 150 images)

**Figure 5.**
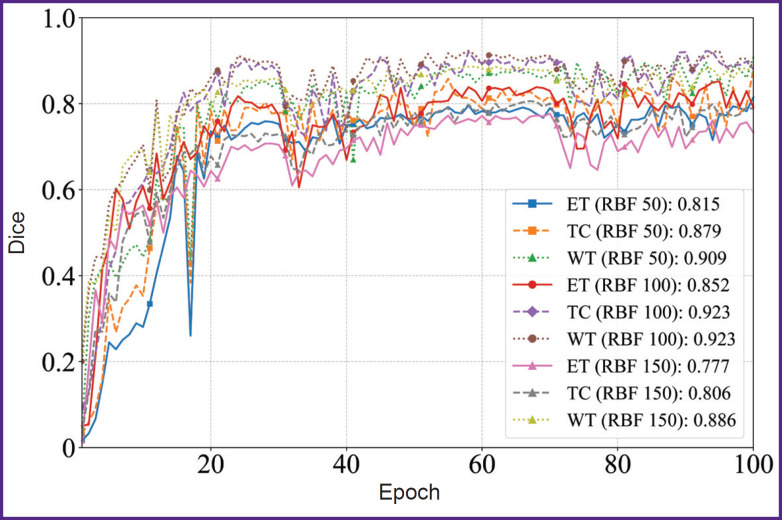
Diagrams of Dice coefficient values depending on the epochs in KANU-Net 2D network for ET, TC, WT classes (50, 100, 150 images)

Figures 4 and 5 illustrate the dynamics of the model training on 50, 100, and 150 images of the sample within 100 epochs. The model showed the stable improvement of quality metrics on the validation sample throughout the training period, achieving the plateau of the mean Dice coefficient after the 60^th^ epoch. The KAN-based model achieved the accuracy comparable with SOTA architectures of convolutional neural networks using smaller volumes of a sample; it suggests the KAN approach applicability to process high-tech medical images.

**Figure 6.**
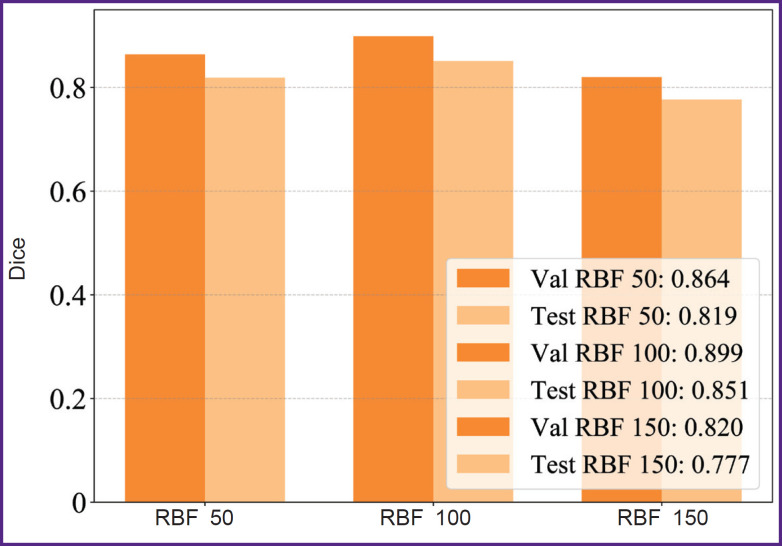
Graphic chart comparing Dice coefficient for validation (val) and test (test) samples of different volumes of data (50, 100, 150 images) in KANU-Net 2D network

Figure 6 represents a comparative diagram of the model performance in different sample volumes on validation and test data. The results demonstrated the sample size n=100 to be the best by achieving Dice coefficient=0.851.

## Discussion

In literature there are the examples of similar studies in MRI segmentation task, however, other authors have used full datasets or large samples of medical images [22], and train the network immediately on several datasets similar in their anatomical structure [23] using heavy three-dimensional images. The study [22] used similar MRI dataset BraTS-GLI 2024 applying KAN model with the modules of effective attention to channels and aggregation of pyramidal features that enabled to achieve Dice coefficient equal to 0.88–0.90 in different tumor regions. At the same time, for the network training there was used a greater number of images: 1080 for a training sample and 135 images — for validation and test samples.

The mean Dice coefficient obtained in the present study (0.851) on the sample containing only 100 images seems to be clinically significant. For a neurosurgeon, such accurate segmentation of the key tumor regions (ET, WT, TC) can be sufficient in certain patient’s management. The Dice value for WT region equal to 0.899 indicated reliable estimability of the total tumor volume. In case of a very large tumor, its eradication can be impossible and due to this it can be inappropriate. In this case, only stereotaxic biopsy is recommended. The approach will enable to improve the preoperative planning quality, especially when determining the surgery volume. In stereotaxic biopsy planning, accurate selection of a contrast accumulating tumor region (ET, Dice=0.812) is critical, since in most cases this particular region corresponds to the most malignant tumor components.

The restriction of the present study of KANU-Net 2D network is the use of only one basic function of RBF, while there is a great variety of them [24], as well as the fixed number (n=4) of grid points (num grids) in the network. These characteristics of KAN network require further research to study medical image segmentation.

The present study, to a greater extent revealed the technical details of using Kolmogorov–Arnold networks to analyze small medical samples; however, its findings have the evident clinical focus. Model training on small datasets will enable to develop specific tools for the segmentation of rare pathologies, when the collection of hundreds of examples is impossible. The implementation of the specified algorithms into clinical practice unlocks capabilities for developing specialized software designed for preoperative image analysis with marking images and tumor volume assessment. Figure 3 represents the visualization of the results, demonstrating the model to adequately cope with the required task, and its prognoses are visually close to the expert marking.

## Conclusion

The research carried out showed KANU-Net 2D to have the precedence over the model for segmentation Med-DANet by both: the mean value and some classes. Moreover, the network reaches the competitive performance on dataset BraTS 2020 compared to other models, as evidenced by mean Dice=0.851 for three tumor regions versus the values 0.852 and 0.855 for nnU-Net and H2NF-Net, respectively. The model efficiency for different tumor regions makes it possible to adapt the KAN-based approach to various image characteristics in medical segmentation tasks.

In addition to technical efficiency, the demonstrated approach has significant clinical potential. Further research should be focused on the model validation under the conditions of real neurosurgical practice and the assessment of segmentation results by practicing neurosurgeons. It will enable to take a step from metrics assessment to the estimation of real benefits for surgical planning.
